# Profiling of extracellular vesicle-associated microRNAs reveals a regulated response to potato virus Y infection in tomato

**DOI:** 10.3389/fgene.2026.1722725

**Published:** 2026-03-09

**Authors:** Lingdie Wang, Xifeng Zhang, Xiang Xu, Shijie Zhang, Jingyuan Ji, Yingwen Wang, Binna Lv, Ying Li, Yubing Jiao, Lili Shen, Jinguang Yang

**Affiliations:** 1 Key Laboratory of Tobacco Pest Monitoring Controlling & Integrated Management, Tobacco Research Institute of Chinese Academy of Agricultural Sciences, Qingdao, Shandong, China; 2 Shanxi Tobacco Company Baoji City Company, Baoji, Shanxi, China; 3 Key Laboratory of National Forestry and Grassland Administration on Tree Genetics and Breeding in the Yellow River Delta, Shandong Academy of Forestry, Jinan, Shandong, China; 4 Qingdao Chengyang District Agricultural and Rural Service Center, Qingdao, Shandong, China

**Keywords:** extracellular vesicles, high-throughput sequencing, miRNA, potato virus Y, virus infection

## Abstract

Plant extracellular vesicles (EVs) serve as critical mediators of intercellular communication during plant-pathogen interactions, particularly through their cargo of regulatory small RNAs, enabling the transport of miRNAs to distant tissues during biotic stress. Potato virus Y (PVY), one of the most economically damaging plant viruses globally, poses significant threats to solanaceous crop production. However, the landscape of EV-associated miRNAs and their regulatory roles in PVY infection remain largely unexplored. In this study, we isolated and characterized EV-associated particles from the apoplastic fluid of both PVY-infected and healthy tomato leaves using differential ultracentrifugation, followed by transmission electron microscopy, nanoparticle size analysis, and western blotting. High-throughput small RNA sequencing revealed 96 significantly differentially expressed miRNAs in EV-associated particles upon viral challenge. Bioinformatic prediction revealed that 80% of these dysregulated miRNAs potentially target multiple genes. Gene Ontology (GO) and Kyoto Encyclopedia of Genes and Genomes (KEGG) pathway analyses demonstrated significant overrepresentation of predicted target genes in pathways associated with transcription, ta-siRNA biogenesis involved in RNA interference, protein binding, RNAi-mediated antiviral immune response, oxidative phosphorylation, mRNA surveillance pathway, and eukaryotic ribosome biogenesis. Our findings demonstrate that PVY infection selectively modulates the miRNA composition within tomato EV-associated particles. These EV-associated particles delivered miRNAs may contribute to a sophisticated antiviral defense mechanism by co-regulating host immunity. This study provides novel insights into the role of EV-associated particles mediated RNA communication in plant immunity and lays a theoretical foundation for developing innovative miRNA- and EV-based antiviral strategies for crop protection.

## 1 Introduction

Extracellular vesicles (EVs) are lipid bilayer-enclosed nanoparticles characterized by a cup-shaped morphology that are capable of transporting diverse cargoes, including proteins, lipids, and nucleic acids (Latifkar et al., 2018). In mammalian systems, EVs are broadly categorized into exosomes, microvesicles, and apoptotic bodies based on their biogenesis pathways and size (Akers et al., 2013; Kalar et al., 2016). Exosomes originate from multivesicular bodies (MVBs) and are typically less than 100 nm in diameter (Akers et al., 2013; Yoshikawa et al., 2019). Microvesicles range from 100 to 1000 nm and are shed directly from the plasma membrane, while apoptotic bodies generally exceed 1000 nm (Yoshikawa et al., 2019; Huang et al., 2021). Although initially identified as a mechanism for disposing of plasma membrane proteins, exosomes and other EVs are now recognized as pivotal players in intercellular communication and the transport of bioactive molecules (Hessvik and Llorente, 2018; Akers et al., 2013).

Similar to mammalian systems, plants secrete multiple EV subtypes that play essential roles in regulating cellular processes and mediating intercellular communication (Bai et al., 2024; U Stotz et al., 2022). Plant EVs are distinguished by specific marker proteins. While markers such as Tetraspanin 8 (TET8), Penetration 1 (PEN1), and Exo70E2 have been well-characterized in *Arabidopsis thaliana*, markers in other species are less defined (Zimmerman et al., 2024; He et al., 2021). Functionally, plant EVs operate analogously to mammalian exosomes by delivering signaling molecules and genetic material to recipient cells (Aryani and Denecke, 2016). For instance, Arabidopsis cells secrete EVs containing small RNAs (sRNAs) that are transferred into fungal pathogens to silence virulence genes (Cai et al., 2018a). Conversely, fungal pathogens can exploit this mechanism to deliver sRNAs into host plants to suppress immunity (Wang et al., 2016).

A growing body of evidence suggests that EVs serve as protective vehicles for the long-distance trafficking of miRNAs, messenger RNAs (mRNAs), and proteins (Yoshikawa et al., 2019). Research has shown that rice-derived EVs can deliver defense proteins into *Rhizoctonia solani* to mitigate fungal infection (Huang et al., 2025). But recent reports have also shown that the majority of plant extracellular RNA (exRNA) is not packaged inside of EVs (Borniego et al., 2025). It is believed that exosomes cannot mediate the intercellular and interorganismal transfer of small RNAs and messenger RNAs (Koch et al., 2025). Among the diverse cargoes, miRNAs are of particular interest due to their essential roles in physiological and pathological processes, including immune responses (Karamanidou and Tsouknidas, 2021). Plant miRNAs, which are typically 19 to 23 nucleotides in length, regulate gene expression through translational inhibition or RNA destabilization (Rey-Burusco et al., 2019; Song et al., 2023). They are pivotal in plant-virus interactions, where they can suppress viral gene expression (Chellappan et al., 2005). Although viruses have evolved suppressors to inhibit host RNA silencing, plants utilize specific defense mechanisms to counteract these viral strategies (Ding and Voinnet, 2007).

Despite the established importance of miRNAs in antiviral immunity, the regulatory dynamics of EV- associated miRNAs during viral infection remain largely unexplored. In this study, we isolated EV-associated particles from both PVY-infected and healthy tomato plants to investigate how viral infection remodels the EV-associated particles miRNA cargo. As the latest research findings indicate that apoplastic RNAs that pellet at 100,000 × g are associated with proteins and/or polysaccharides rather than EVs (Koch et al., 2025). This study did not distinguish between miRNAs originating from EVs and non-EV particles. We identified miRNAs differentially expressed in the apoplastic fluid pellets under 100,000 × g centrifugation following PVY infection. Predictive analyses of target genes indicated that miRNAs present in EV-associated particles are likely involved in regulating antiviral genes and may participate in systemic RNA interference (RNAi). This study highlights the critical role of EV-associated particles as a novel medium for plant-virus interactions and provides a theoretical basis for developing antiviral technologies utilizing miRNA and EV delivery systems.

## 2 Materials and methods

### 2.1 Headings plant materials and virus strain

*Solanum lycopersicum* were cultivated in a greenhouse under the following conditions, with a temperature of 25 °C, a 16/8 h (light/dark) photoperiod, 55% relative humidity, and adequate ventilation. Healthy tomato plants at the 4 to 6 leaf stage were subjected to PVY infection or mock inoculation. Leaf tissue samples were collected 10 days post-treatment. Three biological replicates were collected for each sample group. For PVY infection, infected PVY leaves were ground and diluted 40-fold in phosphate-buffered saline (PBS), followed by mechanical rub-inoculation onto *Solanum lycopersicum* leaves. For the control group, healthy plants were mock-inoculated with 1× PBS. For the PVY-treated group, plants were inoculated with the preserved PVYN:O or PVY-GFP strain. The PVYN:O isolate used in this study is a recombinant strain of Potato virus Y derived from PVYN and PVYO.

### 2.2 Construction of an expression system for the SlyTET8-GFP fluorescently labelled fusion protein

To construct the SlyTET8-GFP vector, the sequence of SlyTET8 was inserted into the psuper1300-GFP expression vector. The recombinant expression vector was then transformed into *Escherichia coli*. Positive clones were selected via single colony screening, and the correct insertion of the SlyTET8 sequence was confirmed by recombinant plasmid sequencing. Purification of recombinant plasmids was accomplished using the EasyPure Plasmid MiniPrep Kit (EM101-02; TransGen Biotech, Beijing, China). Subsequently, the recombinant plasmid was transformed into Agrobacterium, and successful transformants were selected by single colony screening.

The Agrobacterium cells harboring SlyTET8-GFP were resuspended in an infiltration buffer [10 mM MES, 10 mM MgCl_2_, and 200 μM AS], and the OD_600_ was adjusted to 0.8. The bacterial suspension was incubated at room temperature in the dark for 3 h. Tomato leaves were infiltrated using a needle-free syringe for transient overexpression experiments.

### 2.3 Isolation of extracellular vesicles from plant

Extracellular vesicles were isolated from *Solanum lycopersicum* apoplastic wash using a method adapted from the established protocol for *Arabidopsis thaliana* (Huang et al., 2021; Wang et al., 2024). Tomato leaves were harvested and infiltrated with vesicle isolation buffer (20 mM MES hydrate, 2 mM CaCl_2_, 0.1 M NaCl, pH 6.0) under vacuum. The infiltrated leaves were centrifuged at 900 *g* for 10 min at 4 °C to collect the apoplastic fluid. The fluid was centrifuged at 2,000 × g for 30 min at 4 °C to remove cellular debris. The supernatant was subsequently filtered through a 0.45 μm filter. Next, the filtered apoplastic fluid was subsequently centrifuged at 10,000 × g for 30 min at 4 °C. The resulting supernatant was transferred to ultracentrifugation tubes and subjected to ultracentrifugated at 100,000 × g for 2 h at 4 °C. The resulting EV pellet was washed once with PBS and finally resuspended in PBS. For transmission electron microscopy (TEM) observation, the EV sample was loaded onto a 200 mesh copper grid, negatively stained with 1% uranyl acetate for 1 min, and then examined under the microscope.

### 2.4 Nanoparticle size analysis

Nanoparticle size analysis aims to characterise the physical properties of isolated vesicles, confirming that the size distribution of purified particles falls within the typical range for plant-derived EVs. According to the manufacturer’s guidelines, nanoparticle size analysis was performed using a nanoparticle size and zeta potential analyzer. EV samples from both PVY-infected and healthy tomato plants were diluted in PBS to an appropriate concentration for analysis. Three independent measurements were taken for each sample. The acquired data were subsequently processed using the Nanosizer software to determine the particle size distribution.

### 2.5 Western blotting

EV extracts were mixed with an equal volume of RIPA Lysis Buffer supplemented with protease inhibitors and incubated on ice for 30 min, with mixing every 10 min. SDS-loading buffer was added, and equal amounts of samples were loaded onto the lanes of a 12% sodium dodecyl sulfate-polyacrylamide gel electrophoresis (SDS-PAGE) gel for Western blotting. Immunoblot analysis of TET8 was conducted using a rabbit antibody (1:5000), while TET8 protein was detected using a TET8 antibody (1:2000). After transmembrane transfer, antigens were detected by chemiluminescence using an ECL reagent (SuperSignal West Pico Chemiluminescent Substrate kit). The membrane was then visualized using a fluorescence imager, H^+^-ATPase was used as an internal reference for quantitative analysis of band intensity. Three independent experiments were performed for the analyses.

### 2.6 sRNA sequencing and miRNA analysis

Total RNA was extracted from EVs isolated from tomato apoplastic fluid for the construction of an sRNA library. sRNAs were extracted from the samples using the Illumina TruSeq Small RNA Preparation Kit (Illumina, San Diego, United States) to construct sRNA libraries (Li et al., 2017). The sRNA libraries were sequenced on an Illumina HiSeq 2000/2500 platform, generating single-end 50 bp reads. Raw reads were subjected to an in-house program, ACGT101-miR (v4.2) to remove adapter sequences, junk reads, low complexity sequences, common RNA families (rRNA, tRNA, snRNA, snoRNA), repeats and sequences shorter than 18 nt or longer than 25 nt. The remaining small RNAs were classified by alignment to mRNA, RFam and Repbase databases then filtered. Unique sequences with length in 18–25 nucleotide were mapped to *Solanum lycopersicum* precursors in miRBase 22.1 by BLAST search to identify miRNAs. Length variation at both 3′ and 5′ ends and one mismatch inside of the sequence were allowed in the alignment. The unique sequences mapping to *Solanum lycopersicum* mature miRNAs in hairpin arms were identified as known miRNAs. The unique sequences mapping to the other arm of known *Solanum lycopersicum* hairpin opposite to the annotated mature miRNA-containing arm were considered to be novel 5p- or 3p-derived miRNA candidates. The remaining sequences were mapped to other selected species precursors (with the exclusion of *Solanum lycopersicum*) in miRBase 22.1by BLAST search, and the mapped pre-miRNAs were further BLASTed against the *Solanum lycopersicum* genomes to determine their genomic locations. The unmapped sequences were BLASTed against the specific genomes, and the hairpin RNA structures containing sequences were predicated from the flank 120 nt sequences using RNAfold software (http://rna.tbi.univie.ac. at/cgi-bin/RNAfold.cgi). For miRNA differential expression analysis, a p-value <0.05 was set as the threshold for statistical significance.

### 2.7 Differential expressed miRNA targets gene prediction and functional enrichment analysis

GSTAr (Generic Small RNA-Transcriptome Aligner) was used for plant miRNA target prediction through small RNA-transcriptome alignment (Addo-Quaye et al., 2009; Fan et al., 2015). GO and KEGG pathway enrichment analyses were performed using the clusterProfiler R package (v4.x), which implements the hypergeometric test for over-representation analysis (Xu et al., 2024).

### 2.8 Analysis of miRNA expression

Total RNA was extracted from EVs using Trizol reagent (Vazyme, Nanjing, China). Reverse transcription was performed using the TranScript miRNA First-Strand cDNA Synthesis Kit (TransGen Biotech, Beijing, China). Six differentially expressed miRNAs were selected for qRT-PCR validation. The specific miRNA sequences were used as forward primers, while the universal reverse primer was provided in the TranScript miRNA First-Strand cDNA Synthesis Kit (TransGen Biotech, Beijing, China). 5S rRNA was used as the internal reference control standard Ct values. The primer sequences are provided in Supplementary Table S1. The qRT-PCR reactions were run on the ABI 7500 platform, and the relative expression levels of miRNAs were calculated using the 2^−ΔΔCT^ method. Each experiment consisted of three biological and technical replicates.

## 3 Result

### 3.1 Isolation and characterization of EVs from tomato leaves

**FIGURE 1:**
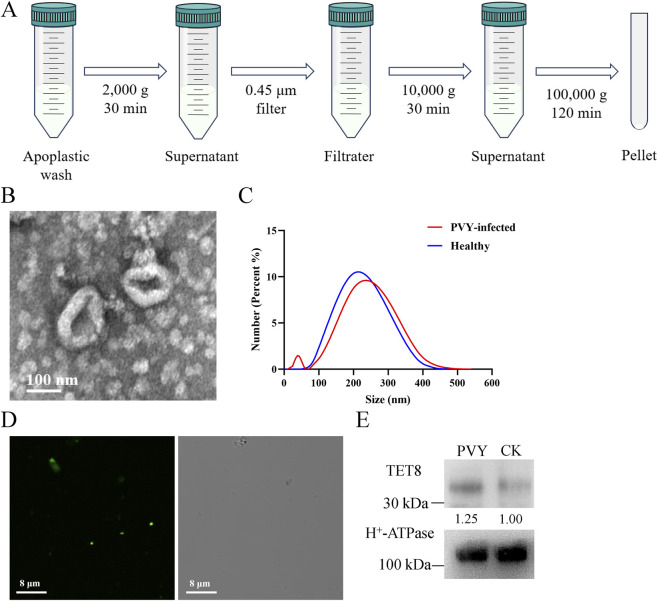
The image shows the various steps of plant EV isolation and identification. **(A)** A protocol for isolating EVs from tomato apoplastic fluid using differential ultracentrifugation. **(B)** Transmission electron microscopy image of isolated tomato EVs. **(C)** Analysis of the size distribution of EVs in healthy and PVY-infected tomato. **(D)** Confocal microscopy images of EVs isolation by ultracentrifugation from apoplastic fluid of SlyTET8-GFP plants. **(E)** The presence of SlyTET8 proteins in EV-associated particles are shown in a Western blot.

EVs were isolated from the apoplastic fluid of tomato leaves through differential ultracentrifugation according to the method applied in Arabidopsis (Figure 1A) (Huang et al., 2021). These EVs appeared as cup-shaped structure under a transmission electron microscopy (Figure 1B). Nanoparticle size analysis showed that EVs derived from PVY-infected and healthy tomato leaves predominantly ranged from 100 to 300 nm in diameter (Figure 1C). The size distribution conforms to the characteristic dimensions of plant extracellular vesicles. Current research indicates that TET8 can serve as a marker for *Arabidopsis thaliana* EVs (He et al., 2021). Through phylogenetic analysis and protein sequence alignment, we identified the homologue of Arabidopsis TET8 in tomato, designated SlyTET8, exhibiting 70.97% sequence homology (Supplementary Figure S1). Its strong enrichment in purified tomato EVs further confirms its validity as a reliable positive marker for tomato extracellular vesicles. We identified the tomato homolog, SlyTET8. We generated expressing fluorescence-tagged fusion proteins SlyTET8-GFP. Confocal microscopy observation of SlyTET8-GFP EVs isolated from tomato apoplastic fluid (Figure 1D). SlyTET8 was detectable in the isolated EVs by Western blot (Figure 1E).

### 3.2 Analysis of sRNA expression profiles in EV-associated particles of PVY-infected *Solanum lycopersicum*

**FIGURE 2:**
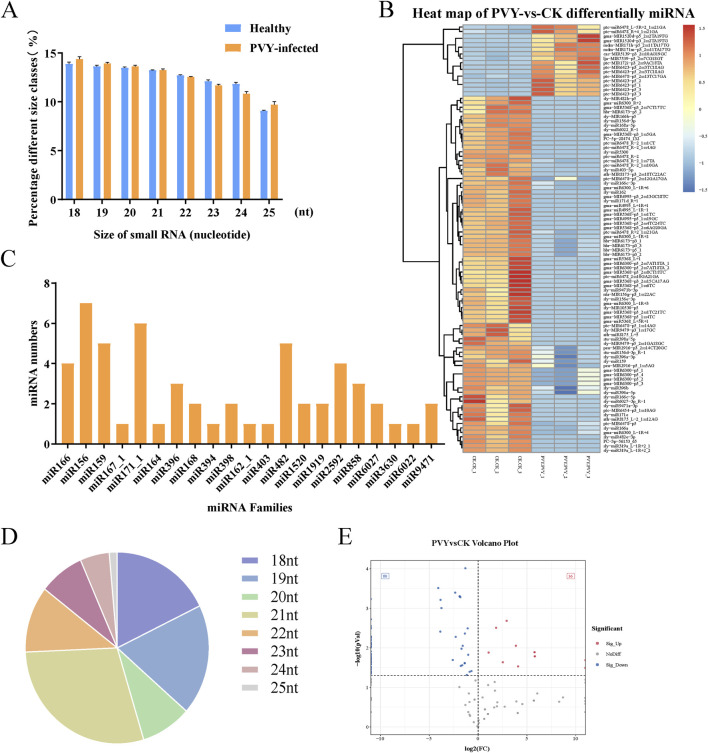
Characterization of miRNA in EV-associated particles from healthy and PVY-infected tomatoes. **(A)** The size distribution of sRNAs in EV-associated particles from healthy and PVY-infected tomatoes. **(B)** Quantitative distribution of conserved miRNA families in sequencing data. **(C)** Proportional length distribution of differentially expressed miRNAs. **(D)** Distribution of differential miRNA numbers. Volcano diagram of the differential miRNAs. The gray dots designate the genes with no remarkable difference. The red and blue dots designate the up- and downregulated miRNAs, respectively, selected according to |log_2_FC|>1 along with a corrected p < 0.05. **(E)** The heat map shows significant differences (p < 0.05) in miRNAs within EV-associated particles under PVY infection. Red indicates higher miRNA expression levels, while blue indicates lower expression levels. CK: Control group.

Numerous studies have demonstrated that plants activate defense responses upon viral infection, subsequently triggering a series of physiological and biochemical changes (Wang et al., 2025). sRNAs, particularly miRNAs, have become a research focus due to their crucial roles in the regulatory networks of plant development (Lozano-Durán, 2024). To further investigate the alterations in the sRNA expression profiles encapsulated within EV-associated particles following PVY infection, we isolated EV-associated particles from both healthy and PVY-infected tomato leaves and performed sequencing with three biological replicates. Current studies have found that sRNAs and longer RNAs are present only in high-density fractions and were absent from EV-enriched low- and medium-density fractions (Koch et al., 2025). Some sRNAs are secreted in complex with ARGONAUTE proteins independent of EVs (Koch et al., 2025). The data sources presented by this research do not distinguish between EVs and non-EV particles. The results showed that the sRNAs ranged from 18 to 25 nt, with predominantly 18, 19, 20 and 21 nt,and the size distributions of sRNAs were essentially identical for the two groups (Figure 2A). Supplementary Table S2 is the summary of read data produced by small RNA sequencing. Statistics were performed on the number of unique reads. The counts of rRNA, snoRNA, snRNA, and tRNA sequence species are provided in Supplementary Table S3. Notably, we detected a total of 21 miRNA families, with the number of miRNAs varying per family. The miR156 family contained the highest number of miRNAs (7 members), followed by the miR171_1 family, which comprised 6 miRNAs, with one-third of these miRNA families contained only a single miRNA member (Figure 2B).

### 3.3 Accumulation of miRNAs in EV-associated particles

To comprehensively investigate the miRNAs carried by EV-associated particles following PVY infection, we analyzed the high-throughput sequencing data of miRNAs. The highest percentage of miRNAs lengths identified were 21 nt and 19 nt, accounting for 28.97% and 18.69% of the total (Figure 2C). Compared to the control group, the miRNA expression profiles after PVY infection exhibited significant alterations. Sequencing results revealed that a total of 179 miRNAs were identified in EVs from both groups, among which 16 miRNAs were upregulated and 80 miRNAs were downregulated (Figure 2D). The expression patterns of these differentially expressed miRNAs are presented in Figure 2E.

**FIGURE 3:**
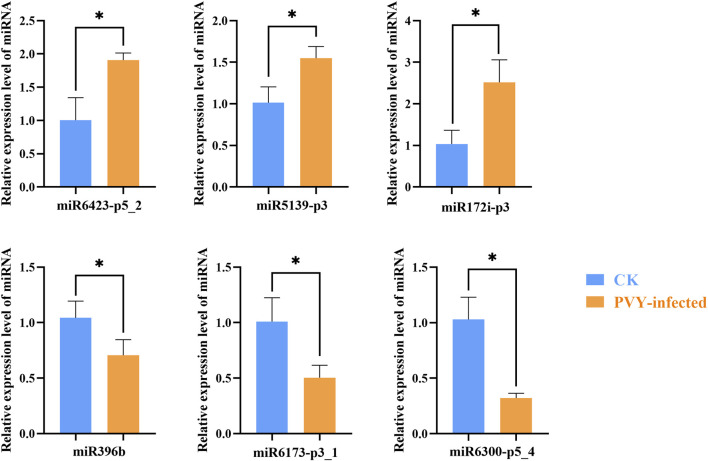
qRT-PCR experiments confirmed the consistency of specific miRNA expression levels, which aligned with the sequencing results. This figure illustrates the relative expression of miRNAs in EV-associated particles from both healthy and PVY-infected tomatoes. The blue bar represents EVs from healthy tomatoes, while the orange bar represents EV-associated particles from PVY-infected tomatoes. The data shown represent the mean ± SD of three biological replicates (n = 3). Statistical significance was determined by t-test, with *p < 0.05. CK: Control group.

Total RNA was extracted from tomato leaves samples and EV-associated particles samples, and qRT-PCR was employed to validate the expression levels of the differentially expressed miRNAs identified by sequencing. The qRT-PCR analysis revealed an upregulation of miR6423-p5_2, miR5139-p3 and miR172i-p3, alongside a downregulation of miR396b, miR6173-p3_1 and miR6300-p5_4, in tomato leaf EVs following PVY infection (Figure 3). A concordant expression level of these miRNAs was observed when comparing the qPCR and sequencing results. Thus, we elucidated the miRNA composition of tomato leaves EV-associated particles and demonstrated that their accumulation patterns vary among different miRNA families. However, the expression levels of these miRNAs in tomato leaves do not entirely correlate with the expression levels of vesicle-associated particles. (Supplementary Figure S2). While the expression trends of certain miRNAs, such as the upregulated miR172i-p3 and the downregulated miR6173-p3_1 and miR6300-p5_4, were consistent between leaf tissues and EVs, we observed significant divergences in others. Notably, miR396b was significantly upregulated in leaves yet downregulated in EVs, whereas miR5139-p3 exhibited downregulation in leaves but upregulation in EVs. Some studies have indicated that exosomes mediate the intercellular and interorganismal transfer of small RNAs and messenger RNAs (Cai et al., 2018b). Meanwhile, other research has shown that miRNAs do not coexist with exosomes (Koch et al., 2025). The functional significance of these EV-associated particles remains unclear. We hypothesize these discrepancies as evidence that the loading of miRNAs into extracellular vesicles is not a passive outcome of cytoplasmic abundance but rather a highly regulated sorting mechanism. This suggests that during PVY infection, the host plant or the virus actively modulates the export of specific miRNAs to distinct cellular compartments to fulfill unique regulatory roles. Furthermore, whether this selective accumulation is influenced by miRNA sequence, target specificity, or other factors awaits further investigation.

### 3.4 Prediction and functional characterization of potential target genes of miRNAs

We analyzed the biological functions of the target genes corresponding to the differentially expressed miRNAs to identify the key genes involved in the miRNA-mediated interactions facilitated by the selective sorting of EV-associated particles during PVY infection. The analysis indicated that among the 96 differentially expressed miRNAs, 15 miRNAs exhibited complementarity to 85 potential target sequences, encompassing a total of 99 binding sites. Among these, only 3 miRNAs were predicted to target a single gene, while 80% of the miRNAs targeted multiple genes (Supplementary Table S4). The miRNA-target gene predictions suggest that these miRNAs are likely to play significant roles in the PVY infection process. Most studies on the function of EV-mediated sRNA transport in mammals have primarily focused on intercellular communication (Umbach and Cullen, 2009). Research on plant EVs has demonstrated that sRNAs encapsulated in exosomes secreted by *Arabidopsis thaliana* can be delivered into fungal cells to silence virulence-related genes (He et al., 2021).

**FIGURE 4:**
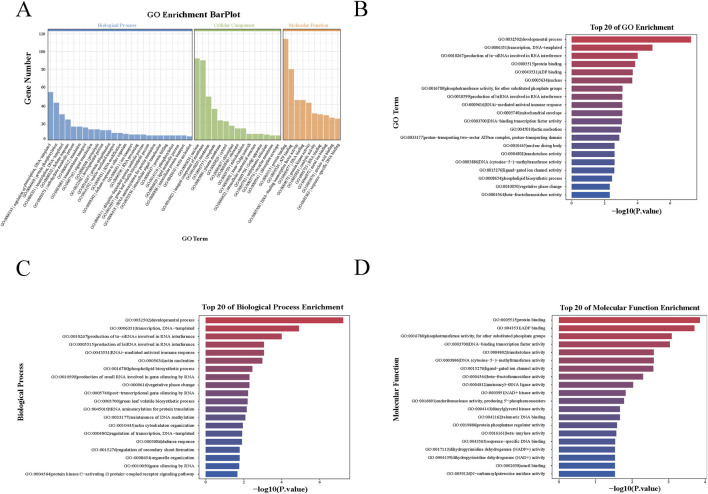
Target gene functions predicted by GO and KEGG enrichment analysis. **(A)** Three GO categories, biological process (BP), cellular component (CC), and molecular function (MF), were selected for functional annotation. **(B)** Top 20 of GO enrichment. **(C)** Top 20 of biological process enrichment. **(D)** Top 20 of molecular function enrichment.

Subsequently, enrichment analysis was performed at the gene level on the targets of the differentially expressed miRNAs in EV-associated particles and tomato leaves during PVY infection to gain functional insights into these miRNAs. We conducted a Gene Ontology (GO) classification analysis on the predicted miRNA targets, across the three standard categories: Molecular Function (MF), Cellular Component (CC), and Biological Process (BP), to evaluate their potential functions. The GO enrichment analysis of EV-associated particles is shown in Figure 4A. The targets of the top 20 specifically expressed miRNAs with the smallest p-values were significantly enriched in processes and functions such as transcription, ta-siRNA biogenesis involved in RNA interference, protein binding, RNAi-mediated antiviral immune response, DNA-binding transcription factor activity, and miRNA biogenesis involved in RNA interference (Figure 4B). The top 20 specifically expressed miRNAs with the smallest p-values were classified based on GO terms related to BP (Figure 4C) and MF (Figure 4D). The GO enrichment analysis of leaves is shown in Supplementary Figure S3. Specifically, among the top 20 specifically expressed miRNA targets in leaves, significant enrichment was observed for processes such as ADP binding, defense response, protein binding, developmental process, ATP binding, and potassium ion transmembrane transporter activity (Supplementary Figure S3).

**FIGURE 5:**
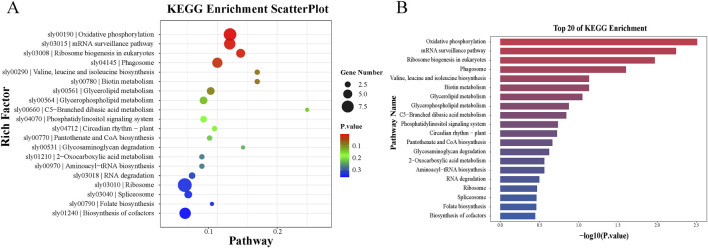
Enriched KEGG pathways of miRNA target genes. **(A)** KEGG enrichment scatterplot. The Rich factor is the ratio of differentially expressed gene numbers annotated in this pathway term to all gene numbers annotated in this pathway term. The P-value is a corrected p-value ranging from 0 to 1, with lower values indicating greater intensiveness. The circle represents the number of enriched genes, with a larger number indicating more enriched genes. **(B)** Top 20 of KEGG enrichment.

Subsequently, Kyoto Encyclopedia of Genes and Genomes (KEGG) pathway enrichment analysis was performed on the target genes. The KEGG pathway enrichment analysis for EV-associated particles is shown in Figure 5A. The results showed that the top 20 specifically expressed miRNAs with the smallest p-values were predominantly enriched in pathways such as oxidative phosphorylation, mRNA surveillance pathway, eukaryotic ribosome biogenesis, phagosome, biotin metabolism, and valine, leucine, and isoleucine biosynthesis (Figure 5B). Furthermore, KEGG pathway analysis of the leaf tissues (Supplementary Figure S4) indicated that target genes were significantly enriched in pathways including RNA degradation, aminoacyl-tRNA biosynthesis, ubiquitin-mediated proteolysis, phagosome, butanoate metabolism, monobactam biosynthesis, RNA polymerase, and plant hormone signal transduction. The prediction analysis of potential miRNA target genes suggests that the miRNAs carried by EV-associated particles may be directly involved in regulating the expression of antiviral-related genes and may contribute to antiviral defense through vesicle-mediated participation in the RNA interference pathway.

## 4 Discussion

Elucidating the molecular dialogue between plants and viruses is pivotal for understanding antiviral immunity (Borges and Martienssen, 2015). While intracellular miRNA dynamics during viral infection have been extensively characterized (Ammara et al., 2015), the role of e EV-associated particles miRNAs remains a nascent field (Moulin et al., 2023). In this study, we demonstrate that PVY infection triggers a profound remodeling of the miRNA cargo within tomato extracellular fluid. These changes exhibit specific patterns that do not entirely correspond to the miRNA content within tomato leaves. These findings imply the existence of a selective and potentially regulated process for miRNA sorting into the extracellular space, which may play a role in modulating systemic defense responses.

A striking feature of our data is the predominance of 21-nt small RNAs within the EVs. In plants, 21-nt sRNAs are typically associated with ARGONAUTE 1 (AGO1) and are the primary effectors of post-transcriptional gene silencing (Mi et al., 2008). The specific enrichment of this size class, rather than a random distribution of RNA fragments, implies that the RNA into tomato extracellular fluid is governed by specific RNA-binding proteins. This aligns with findings in Arabidopsis, where RNA-binding proteins such as ANNEXIN1 and AGO1 were found to selectively stabilize and load sRNAs into EVs (He et al., 2021). Some studies have also reported that the plant leaf apoplast contains abundant and diverse species of RNA outside EVs, which are protected from RNase degradation by a group of extravesicular RNA-binding proteins (Zand Karimi et al., 2022). In our study, miRNAs originating from vesicular and non-vesicular particles were not distinguished. Therefore, these differentially expressed miRNAs may originate from either exosomes or non-vesicular sources. The association between these miRNAs and vesicles, as well as their functional mechanisms, requires further investigation.

Our differential expression analysis revealed a significant skew toward miRNA downregulation following PVY infection, with 80 miRNAs repressed and only 16 induced. This phenomenon warrants deep consideration. We hypothesize two potential biological scenarios driving this trend. First, it may represent a viral counter-defense strategy where PVY suppresses the biogenesis or export of host miRNAs to prevent the spread of silencing signals to neighboring tissues. Viruses are known to deploy silencing suppressors to inhibit host RNAi machinery, and blocking EV-mediated transport could be an extension of this virulence mechanism (Csorba et al., 2009). Alternatively, this downregulation might reflect a host retentive strategy. Upon infection, the plant cell may prioritize intracellular defense by retaining immune-related miRNAs within the cytoplasm to combat the replicating virus directly, thereby reducing the pool available for extracellular export (Silhavy and Burgyán, 2004).

Despite the general trend of downregulation, the specific upregulation of select miRNAs, such as miR6423 and miR5139, suggests they play unique roles in the interaction. The functional enrichment analysis of their target genes highlights the involvement of the mRNA surveillance pathway and RNAi-mediated antiviral responses (Borges and Martienssen, 2015). This is particularly intriguing because the mRNA surveillance pathway is critical for detecting and degrading aberrant viral transcripts (Conti and Izaurralde, 2005). We postulate that EV-associated particles delivered miRNAs may prime uninfected neighboring cells by downregulating negative regulators of immunity, thereby establishing a pre-activated antiviral state ahead of the spreading infection. This mechanism would function analogously to the systemic acquired resistance signaling observed in plant-fungal interactions, yet tailored for the intracellular nature of viral pathogens (Spoel and Dong, 2008).

Furthermore, our findings bridge a gap in understanding how plants manage viral infections compared to fungal pathogens. While previous studies have shown that plants export sRNAs into fungal cells to silence virulence genes (Cai et al., 2018a), during viral infection, these extracellular vesicle-associated miRNAs primarily function to mediate intercellular communication and regulate gene expression involved in systemic defence, rather than directly targeting pathogens. Since viruses lack cellular structure, EV-associated particles cannot fuse with the pathogen; instead, they likely fuse with adjacent plant cells (Moulin et al., 2023). Specifically, EVs encapsulated with specific miRNAs are secreted into the apoplast and can be taken up by neighboring or distal recipient cells. Once internalized, the released miRNAs can suppress the expression of target genes to systemically regulate plant immunity. PVY infection actively remodels the EV cargo to either manipulate host defenses or, conversely, that the host utilizes EVs to transmit antiviral silencing signals to uninfected tissues. The precise mechanisms underlying this bidirectional interplay remain unclear, and future studies aimed at identifying the viral and host factors involved in vesicle cargo sorting will be essential to resolving this critical question in plant-virus interactions. Tomato EV-associated particles miRNA likely function as endogenous messengers that coordinate multicellular resistance. The presence of these miRNAs in the apoplastic space, particularly within the EV-enriched fraction which is capable of crossing cell walls, suggests their potential role as mobile signaling molecules during plant-virus interaction. Whether they are transported via exosomes, non-vesicular complexes, or both, their differential accumulation in this extracellular compartment implies a regulated secretion process triggered by PVY.

In summary, our miRNA-seq analysis of apoplastic EV-enriched fractions from PVY-infected tomato leaves has revealed a distinct set of differentially expressed, extracellular miRNAs. Although the exact carrier (exosomal or non-vesicular) for each miRNA remains to be elucidated, their altered abundance in a fraction known to participate in intercellular communication highlights their potential as key regulators in the systemic landscape of host-pathogen interaction. These findings challenge the traditional view of cell-autonomous antiviral RNAi and point toward a sophisticated intercellular communication network (Borges and Martienssen, 2015). This study establishes a foundation for future research to dissect the precise transport mechanisms and functional validation of these candidate signaling miRNAs. Such insights could lead to the development of bioengineered EVs as delivery vectors for RNA-based antiviral therapeutics in agriculture (Mitter et al., 2017).

## Data Availability

The original contributions presented in the study are publicly available. This data can be found at the Genome Sequence Archive (Genomics, Proteomics & Bioinformatics 2025) in National Genomics Data Center (Nucleic Acids Res 2025), with the accession number CRA038241, https://ngdc.cncb.ac.cn/gsa/browse/CRA038241.
