# Supplementary material for: Profiling of extracellular vesicle-associated microRNAs reveals a regulated response to potato virus Y infection in tomato
Source: Front Genet. 2026 Mar 9;17:1722725. doi: 10.3389/fgene.2026.1722725 (PMC13006145; doi:10.3389/fgene.2026.1722725)
Supplement: Supplementary file 3 [file Supplementaryfile1.docx]

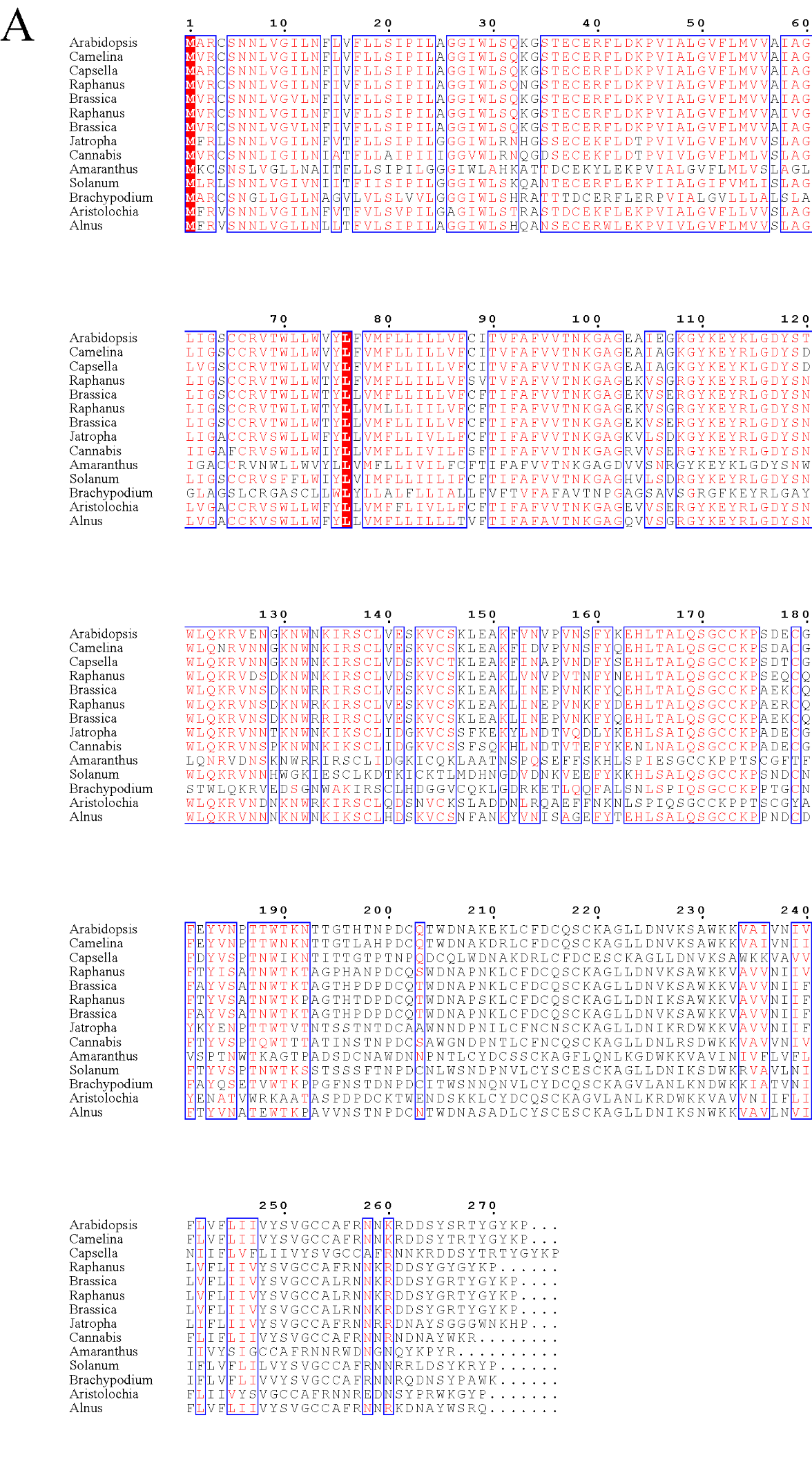


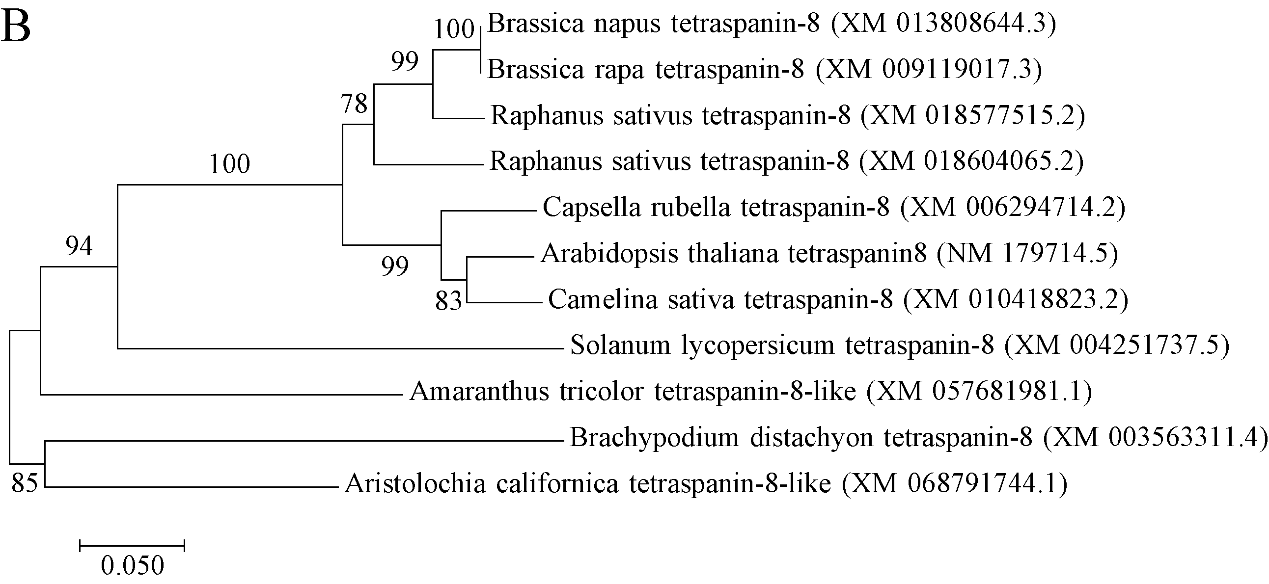


**Supplementary Figure S1.** Protein sequence alignment and phylogenetic analysis of *Arabidopsis thaliana*, *Solanum lycopersicum*, and other representative plants. (A) Protein sequence alignment analysis of *Arabidopsis thaliana*, *Solanum lycopersicum*, and other representative plants. 14 plant species were selected for protein sequence alignment, exhibiting homology with *Arabidopsis thaliana* at 92.46%, 89.89%, 83.69%, 82.50%, 82.55%, 82.50%, 72.16%, 72.30%, 71.88%, 70.97%, 80.82%, 77.13%, and 93.33%. (B) Phylogenetic analysis of *Arabidopsis thaliana*, *Solanum lycopersicum*, and other 11 representative plants.


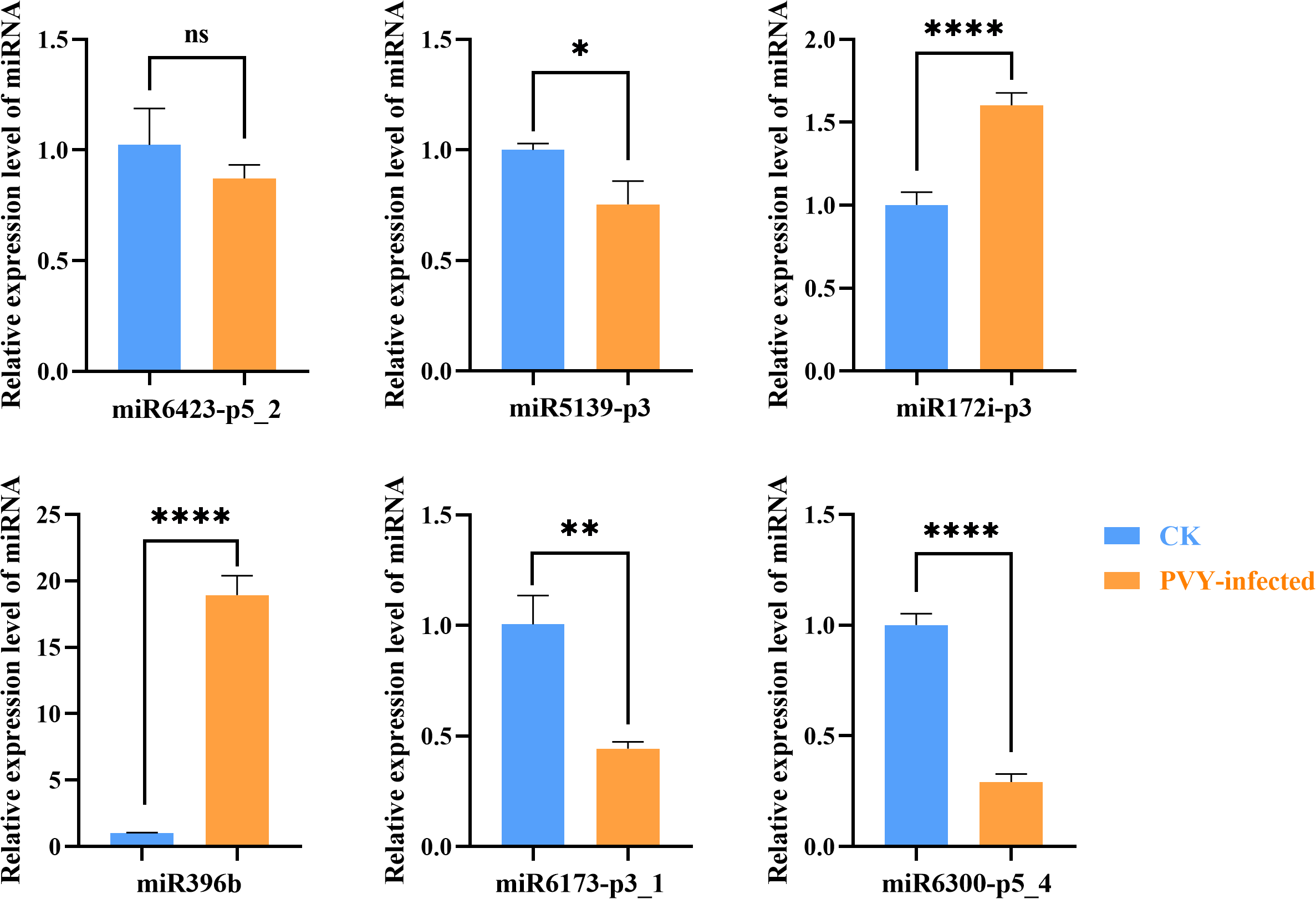


**Supplementary Figure S2.** qRT-PCR experiments were conducted to detect the relative expression levels of miRNAs in tomato leaves. Blue bars represent expression levels in healthy tomato leaves, while orange bars denote expression levels in PVY-infected tomato leaves. Data presented represent the mean ± standard deviation from three biological replicates (n=3). Statistical significance was determined using t-tests, with *p < 0.05, **p < 0.01 and ****p< 0.0001. CK: control group.


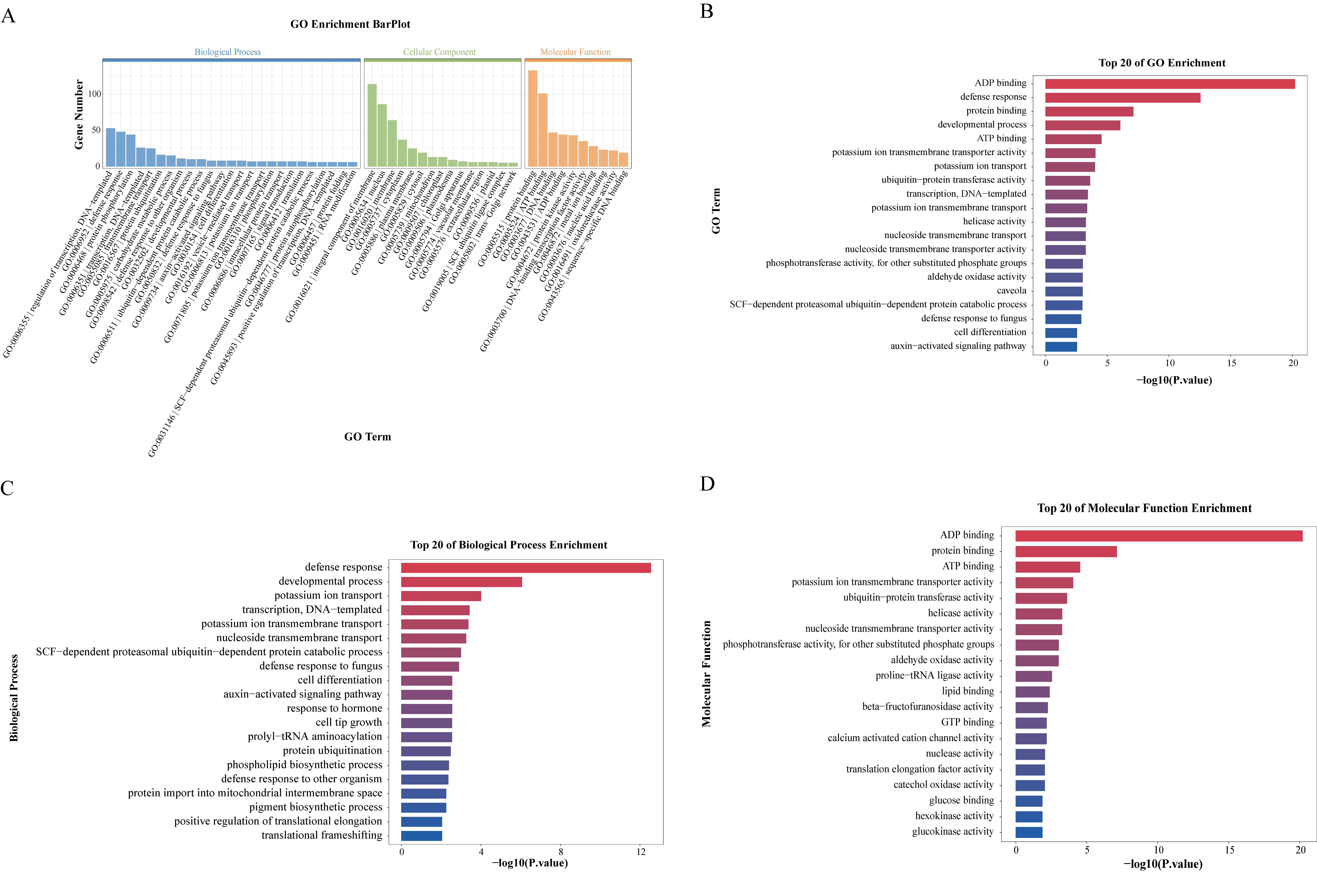


**Supplementary Figure S3.** Target gene functions predicted in tomato leaves by GO enrichment analysis. (A) Three GO categories, biological process (BP), cellular component (CC), and molecular function (MF), were selected for functional annotation. (B) Top 20 of GO enrichment. (C) Top 20 of biological process enrichment. (D) Top 20 of molecular function enrichment.


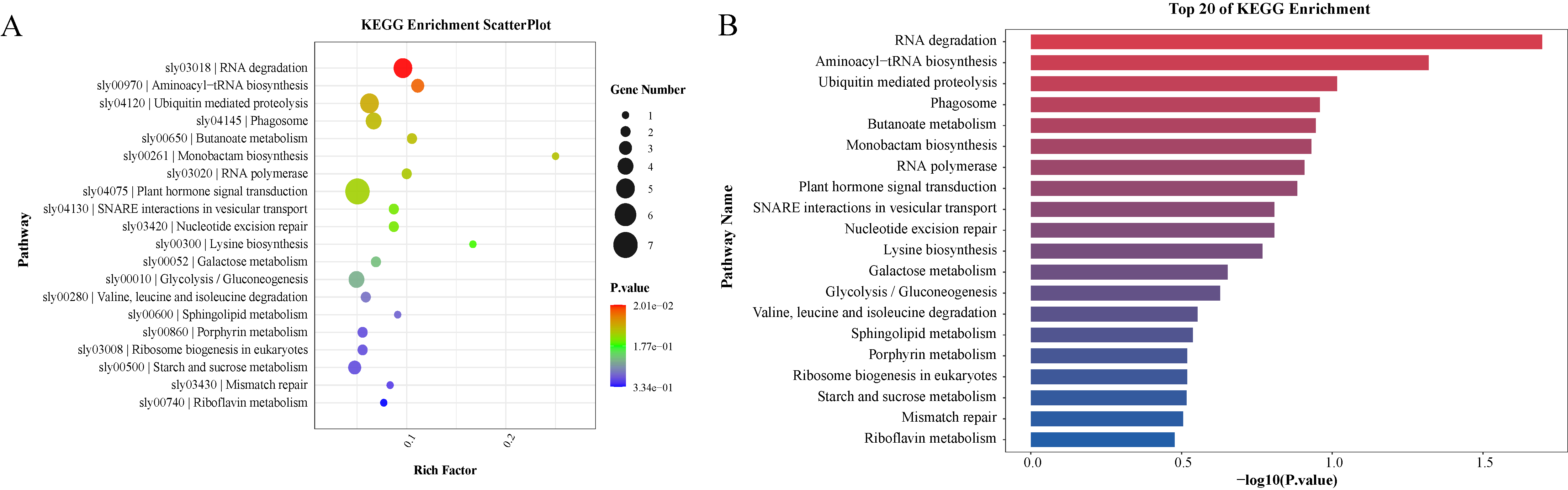


**Supplementary Figure S4.** Enriched KEGG pathways of miRNA target genes in tomato leaves. (A) KEGG enrichment scatterplot. The Rich factor is the ratio of differentially expressed gene numbers annotated in this pathway term to all gene numbers annotated in this pathway term. The P-value is a corrected p-value ranging from 0 to 1, with lower values indicating greater intensiveness. The circle represents the number of enriched genes, with a larger number indicating more enriched genes. (B) Top 20 of KEGG enrichment.
